# Hepcidin and Iron Homeostasis in Patients with Subacute Thyroiditis and Healthy Subjects

**DOI:** 10.1155/2019/5764061

**Published:** 2019-02-27

**Authors:** Aleksandra Hernik, Ewelina Szczepanek-Parulska, Dorota Filipowicz, Agata Czarnywojtek, Elżbieta Wrotkowska, Lucyna Kramer, Alina Urbanovych, Marek Ruchała

**Affiliations:** ^1^Department of Endocrinology, Metabolism and Internal Medicine, Poznan University of Medical Sciences, Poland; ^2^Department of Computer Science and Statistics, Poznan University of Medical Sciences, Poland; ^3^Department of Endocrinology, Lviv National Medical University, Ukraine

## Abstract

**Purpose:**

Hepcidin is an acute-phase protein involved also in regulation of iron homeostasis. The aim of the study was to prospectively assess for the first time the hepcidin_EL_ concentration in patients with subacute thyroiditis (SAT), to identify biochemical determinants of hepcidin_EL_ concentration and evaluate the potential role of hepcidin in SAT diagnosis and monitoring.

**Methods:**

Out of 40 patients with SAT initially recruited, restrictive inclusion criteria fulfilled 21 subjects aged 45 ± 10 years and 21 healthy control subjects (CS). Hepcidin_EL_ concentration, thyroid status, and iron homeostasis were evaluated at SAT diagnosis and following therapy and compared with CS.

**Results:**

The median hepcidin_EL_ concentration at SAT diagnosis is higher than that in CS (48.8 (15.9-74.5) ng/mL vs. 18.2 (10.2-23.3) ng/mL, *p* = 0.009) and is significantly lower after treatment (4.0 (1.2-10.0) ng/mL, *p* = 0.007) compared with CS. The ROC analysis for hepcidin_EL_ at SAT diagnosis revealed that area under the curve (AUC) is 0.735 (*p* = 0.009), and the cut-off for hepcidin_EL_ concentration is 48.8 ng/mL (sensitivity 0.52 and specificity 0.95). Hepcidin_EL_ in SAT patients correlated with CRP (*r* = 0.614, *p* = 0.003), ferritin (*r* = 0.815, *p* < 0.001), and aTPO (*r* = -0.491, *p* = 0.024). On multiple regression, the correlation between hepcidin_EL_ and ferritin was confirmed (*p* < 0.001).

**Conclusions:**

SAT is accompanied by a significant increase in hepcidin, which reflects an acute-phase inflammatory process. Parameters of iron homeostasis improved significantly while inflammatory indices got lower following recovery. The potential role of hepcidin as a predictive factor of the risk of SAT relapse needs to be assessed in studies on larger groups of SAT patients.

## 1. Introduction

**Figure 1:**
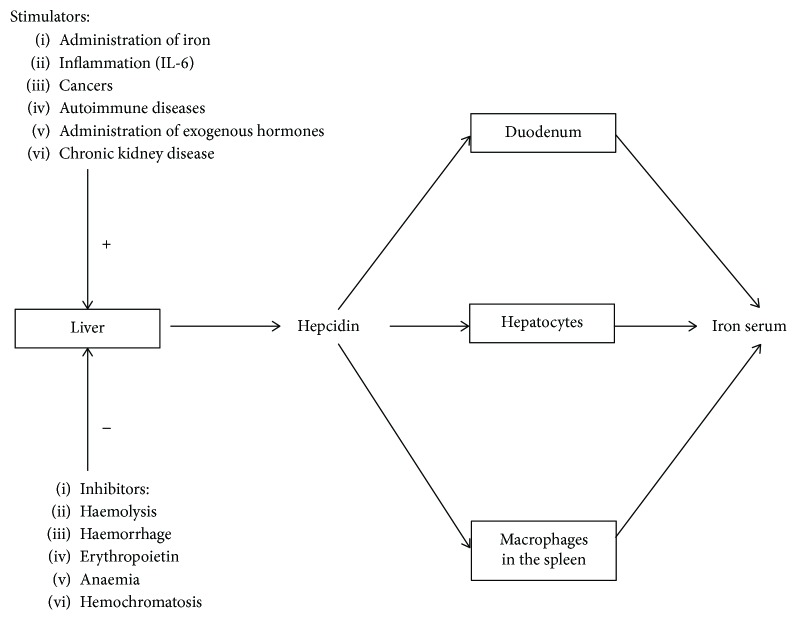
The stimulators and inhibitors of hepcidin in iron homeostasis. The clinical factors and level of iron in the serum impact the secretion of hepcidin. The stimulators increase concentration of hepcidin, inhibiting iron transfer from the lumen of the duodenum to the serum, from ion storage in the hepatocytes and macrophages located in the spleen. The inhibitors act antagonistically.

Hepcidin is a protein hormone produced by hepatocytes, responsible for the regulation of systemic and local iron (Fe) concentration [1]. It acts by direct influence on ferroportin, which is a transporter protein carrying Fe ions from the lumen of the duodenum to the serum [2]. Interaction of hepcidin with ferroportin leads to inactivation of the latter, which results in reduction of the serum Fe level [3]. Moreover, hepcidin influences the process of Fe storage in the liver cells and impacts macrophages of the spleen to stimulate the erythrocyte degradation [4, 5]. Thus, a chronically reduced level of hepcidin may lead to Fe overload, while its excess to refractory anaemia [6]. In turn, the high Fe overload stimulates the production of hepcidin [7], as the main physiological role of hepcidin is to prevent from excessive Fe accumulation. Previously published reports have indicated certain conditions, which stimulate or inhibit hepcidin production (Figure 1) [3]. Besides important function in regulation of Fe homeostasis, hepcidin is also an acute-phase protein, which was found to be increased in inflammatory diseases [8].

Another known regulators of erythropoiesis are thyroid hormones acting by direct influence on proliferation of erythrocyte precursors and stimulation of erythropoietin renal production [9]. Both hyper- and hypothyroidism have been found to increase the risk of accompanying disturbances in Fe balance and anaemia [10]. On the other hand, Fe deficiency may adversely affect thyroid hormone status, as Fe is vital for the activity of thyroid peroxidase, crucial in the first steps of thyroid hormone synthesis [11]. In recent studies, several red blood cells (RBC) indices have been demonstrated to correlate with thyroid function [12–14].

The mutual interplay between Fe homeostasis and thyroid status is an important issue to be evaluated in a clinical setting. Two recent studies have evaluated the hepcidin concentration in the context of thyroid function. Hepcidin_EL_ (measured by ELISA) concentration was to date assessed in patients with newly diagnosed hyperthyroidism (in vast majority due to Graves' disease) and following restoration of euthyroidism; however, no significant differences in its concentration were observed [15, 16]. To the best of our knowledge, hepcidin has never been assessed in a large well-characterized group of patients with subacute thyroiditis.

Subacute thyroiditis (SAT) is a rare type of thyroiditis, affecting women 3-5 times more frequently than men, usually in their middle age [17–19]. An average incidence of SAT is 5 : 100,000 per year, while SAT patients constitute less than 5% of patients with thyroid disorders [17, 19]. The etiopathogenesis of the disease is still unclear; however, viral infections occurring prior to the disease development are thought to be the trigger [20]. Nevertheless, certain genetic factors and autoimmune component are also postulated to predispose to the development of SAT [21]. The disease is characterized by severe pain in the anterior neck region, diffuse goitre, characteristic ultrasound picture, fever, raised inflammatory markers such as C-reactive protein (CRP), and typical fluctuations of thyroid hormones [22]. On diagnosis, most patients present with transient hyperthyroidism due to destruction of thyrocytes and release of thyroid hormones, but in 6-8 weeks, majority of them become euthyroid [17]. In some patients, the hypothyroid phase may develop later on, but in majority of them, spontaneous restoration of the euthyroid state occurs in a few months [17]. The combination of thyroid dysfunction and the acute-phase inflammatory process accompanying SAT development prompted us to assess the hepcidin level in this group of patients.

Thus, the aim of our study was to prospectively assess the hepcidin_EL_ concentration and other parameters describing Fe homeostasis in patients with SAT on diagnosis and in the remission phase, compared to heathy control subjects (CS). In addition, we aimed to identify biochemical determinants of hepcidin_EL_ concentration in the course of SAT as well as to make an attempt to evaluate the potential role of hepcidin as a marker useful in the diagnosis and monitoring of SAT.

### 1.1. Subjects and Methods

#### 1.1.1. Study Population

This was a prospective observational study. The subjects consisted of patients newly diagnosed with a first episode of SAT. The patients were enrolled and followed up from January 2016 until June 2018. The control subjects (CS) comprised healthy volunteers matched for age and gender.

The diagnosis of SAT was based on clinical, laboratory, and ultrasound (decreased, inhomogeneous echogenicity on ultrasound examination with accompanying hypovascularity on color Doppler examination) features. The health of CS was certified by clinical features (good general condition, no signs of thyroid disorders), biochemical samples (all parameters assessed in this study depicted in Table 1 within the normal ranges), and normal thyroid ultrasound examination.

**Figure 2:**
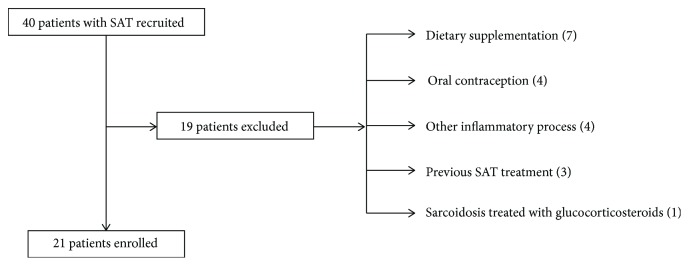
The clinical exclusion criteria of the patient recruitment.

To obtain reliable results, in both groups, strict exclusion criteria were adopted (Figure 2). Numerous factors, which may potentially affect hematopoiesis, and/or the hepcidin level occurring less at the moment of the study or up to 6 months before were disqualified from the study, i.e., dietary supplementation (Fe, vitamin B12, and folic acid) in the previous 6 months, other situations potentially affecting hematopoiesis (haemolysis, haemorrhage, surgical therapy, and symptomatic anaemia), cancer diagnosis, autoimmune disease, acute inflammatory disease (other than SAT), administration of exogenous hormones (oral contraception or hormonal replacement therapy, chronic therapy with glucocorticosteroids, and erythropoietin), pregnancy or breastfeeding, chronic kidney or liver diseases, hemochromatosis, and previous therapy of SAT.

Altogether, 40 patients with SAT diagnosis were initially recruited to the project. However, due to restrictive exclusion criteria, 21 subjects were finally enrolled. The same number of patients constituted the control group of healthy subjects. Eventually, a sample of 21 patients (two of them males) aged between 35 and 72 years (45 ± 10 years) and the CS consisting of 21 subjects (matched for gender) aged between 31 and 70 years (46 ± 11 years) were enrolled for examinations. All postmenopausal women were not qualified.

All patients gave their written informed consent to participate in the study. The project is in accordance with the declaration of Helsinki. The study was approved by the local bioethical committee of the Poznan University of Medical Sciences (approval number: 386/16).

#### 1.1.2. Laboratory Assessment

The following laboratory tests were performed in all patients with SAT and CS enrolled to the study: thyroid-stimulating hormone (TSH), free thyroid hormones (fT3, fT4), anti-thyroid peroxidase antibodies (aTPO), anti-thyroglobulin antibodies (aTG) and anti-TSH receptor antibodies (TRAb), CRP, ferritin, Fe, complete blood count (CBC), creatinine, aminotransferases (ALT, AST), and hepcidin_EL_ concentration in the serum.

Laboratory parameters were assessed in patients at the moment of SAT diagnosis (T0) and achieving biochemical remission (T1) 12 weeks later. On diagnosis, the patients were subjected to prednisolone treatment. The initial dose was 40 mg, tapered by 5 mg a week. The duration of pharmacological therapy was 8 weeks. In the CS, the same laboratory tests were done once. The biochemical parameters of ALT, AST, Fe, creatinine, and CRP were assessed by a Hitachi Cobas e501 analyser (Roche Diagnostics, Indianapolis, USA), and the measurements of thyroid-related parameters (TSH, fT3, fT4, aTPO, aTG) and ferritin were performed using a Hitachi Cobas e601 chemiluminescent analyser (Roche Diagnostics, Indianapolis, IN, USA). The TRAb level was determined by the radioimmunological method with commercially available TRAK RIA kits (BRAHMS GmbH, Hennigsdorf, Germany). The complete blood count was measured by automated flow cytometer Sysmex-XN 1000 (Sysmex Europe GmbH, Bornbarch, Germany). Hepcidin_EL_ was measured by the Hepcidin 25 (bioactive) HS ELISA which is a high sensitive enzyme immunoassay for the quantitative *in vitro* diagnostic measurement (DRG Instruments GmbH, Germany).

#### 1.1.3. Statistical Methods

The acquired data were analysed and presented statistically using STATISTICA software (StatSoft, Tulsa, Oklahoma, USA), and presented figures were performed by Analyse-it Software Ltd. add-in for Microsoft Excel (United Kingdom) and PQStat Software (Poland). Before statistical calculations, normal distribution was tested. The parameters with different reference ranges in men and women such as RBC, haemoglobin (HGB), haematocrit (HCT), Fe, and ferritin were calculated only in the female subgroup. Values are expressed as median and interquartile range (IQR) for nonparametric tests and mean ± standard deviation (SD) for parametric tests. Comparisons of two groups (SAT vs. CS) were done using the nonparametric Mann–Whitney *U* test and parametric, independent *T*-test. The nonparametric Wilcoxon signed-rank test and dependent *T*-test were used to compare two related samples of SAT (T0 vs. T1). Statistical calculation of the receiver operating characteristic (ROC) curve was performed using DeLong's method. Spearman's rank correlation coefficient was used for evaluation of hepcidin_EL_ and every laboratory parameter measured in this study. The univariate analysis was performed for hepcidin_EL_, white blood cells (WBC), CRP, Fe, and ferritin in the group of untreated patients (SAT T0) and in CS. The multiple regression as well as forward and backward stepwise regression analyses were conducted. The level of statistical significance was set at *p* < 0.05.

## 2. Results

### 2.1. Hepcidin

**Figure 3:**
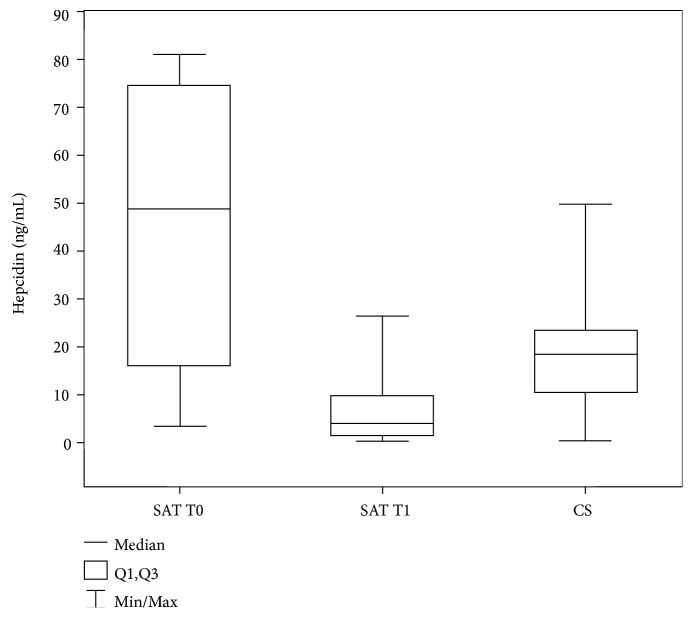
The level of hepcidin_EL_ in patients with subacute thyroiditis (SAT) at the moment of diagnosis (T0), follow-up (T1), and in the healthy control subjects (CS). Values are expressed as median and interquartile range (IQR).

The median of hepcidin_EL_ concentration in the serum of patients with SAT (T0, 48.8 [15.9-74.5] ng/mL) is statistically significantly elevated (*p* = 0.009) in comparison to CS (18.2 [10.2-23.3] ng/mL) and is significantly lower (*p* = 0.007) after treatment (T1, 4.0 [1.2-10.0] ng/mL) than in CS (Tables 1 and 2, Supplementary Table 1, Figure 3).

The ROC analysis for hepcidin_EL_ at T0 shows that the calculated area under curve (AUC) is 0.735 (*p* = 0.009) (Supplementary Figure 1), and according to the Yoden method, the cut-off for hepcidin_EL_ concentration is equal to 48.8 ng/mL. For that value, the sensitivity is 0.52 while specificity is 0.95. The crossing proportion point corresponds to the value equal to 22.5 ng/mL, with the sensitivity of 0.66, while specificity equals to 0.71 (Supplementary Figure 2).

Secondly, the concentration of hepcidin_EL_ T0 was correlated with all assessed blood parameters listed in Table 1, by calculating Spearman's correlation coefficient. A statistically significant correlation is found: hepcidin_EL_ with CRP (*r* = 0.614, *p* = 0.003), hepcidin_EL_ with ferritin (*r* = 0.815, *p* < 0.001), and hepcidin_EL_ with aTPO (*r* = -0.491, *p* = 0.024) (Supplementary Figure 3). The concentration of hepcidin_EL_ was also correlated with the same parameters in the control group, and statistically significant positive correlation is demonstrated for hepcidin_EL_ and ferritin (*r* = 0.837, *p* < 0.001), (Supplementary Figure 4a) as well as for hepcidin_EL_ and Fe (*r* = 0.540, *p* = 0.017), (Supplementary Figure 4b).

Based on earlier reports and own experience, several parameters (hepcidin_EL_, WBC, HGB, CRP, ferritin, and Fe) were selected for univariate analysis. The calculations were performed in two groups: SAT T0 and CS (Supplementary Table 2). In the SAT group, a significant positive correlation is found between hepcidin_EL_ and CRP, as well as hepcidin_EL_ and ferritin. In CS, a significant positive correlation is found between hepcidin_EL_ and ferritin as well as hepcidin_EL_ and Fe.

The common, positive, and statistically significant correlation in both groups (SAT T0 and CS) is found between the concentration of hepcidin_EL_ and ferritin. To verify and confirm observed correlations, the multiple regression (*p*_SAT_ < 0.001, *p*_CS_ < 0.001), forward (*p*_SAT_ = 0.004, *p*_CS_ < 0.001), and backward (*p*_SAT_ < 0.001, *p*_CS_ < 0.001) stepwise regression analyses were performed and confirmed the correlation between hepcidin_EL_ and ferritin. In CS, a statistically significant correlation between hepcidin_EL_ and Fe (*p* = 0.046) is found in multiple regression analysis.

### 2.2. Other Parameters

The level of each laboratory parameter assessed in the study and listed in Table 1 was compared between the SAT T0 and CS. Many of them such as RBC, HGB, HCT, mean corpuscular volume (MCV), mean corpuscular haemoglobin (MCH), mean corpuscular haemoglobin concentration (MCHC), red blood cell distribution width-coefficient of variation (RDW-CV), and Fe are found to be statistically significantly lower in the SAT group if compared to CS. No statistically significant difference between ferritin concentrations is observed between patients with SAT T0 and CS. The platelet (PLT) concentration both in the SAT group and in CS is within the normal range, however is statistically significantly higher at SAT T0 than in CS. Other platelet parameters (PDW (platelet distribution width), MPV (mean platelet volume), and P-LCR (platelet larger cell ratio)) are lower in SAT T0 in comparison to CS. The CRP concentration in the cohort of SAT T0 patients is significantly higher (*p* < 0.001) than that in CS. The ALT, AST, and creatinine concentration do not differ significantly between the SAT T0 group and CS. The SAT T0 patients present biochemical indices of hyperthyroidism (TSH below normal range; fT3 and fT4 above the normal range), while CS are clinically and biochemically euthyroid. SAT patients have normal concentration of TRAb and aTPO, while significantly elevated aTG concentration if compared to CS.

Each laboratory parameter listed in Table 1 was compared for patients with SAT T0 and after remission (T1). The parameters demonstrating statistically significant differences are presented in Table 2. The parameters related to Fe homeostasis (RBC, HGB, HCT, MCH, MCHC, RDW-CV, and Fe) are statistically significantly higher after restoration of normal thyroid function and remission of SAT, while inflammatory indices (WBC, PLT, ferritin, and CRP) are statistically lower following recovery. In addition, PDW, MPV, and P-LCR are higher after treatment in comparison with SAT T0 patients. With the therapy, normalisation of thyroid function tests is observed (increase in the TSH level; decrease of fT4 and fT3). In addition, slight but statistically significantly lower values of TRAb concentration are observed following therapy.

The obtained parameters in SAT T1 and in healthy CS also were compared. The parameters presenting statistically significant difference are presented in the Supplementary Table 1. In patients with SAT T1, the parameters MCV, MCH, and ferritin are statistically significantly lower if compared to CS, while RDV-CV is significantly higher, compared to CS.

## 3. Discussion

This study constitutes the first prospective evaluation of hepcidin_EL_ concentration in patients with SAT and following treatment, in comparison to healthy CS. We investigated also the relationship between hepcidin_EL_ and biochemical parameters reflecting severity of the inflammatory process, thyrometabolic status, and Fe homeostasis.

We have found that hepcidin_EL_ concentration, similarly to CRP, is markedly elevated at diagnosis of SAT and becomes reduced after recovery. The hepcidin was even lower in patients soon after glucocorticosteroid therapy compared to healthy CS, which could be explained with strongly immunosuppressive action of prednisolone, despite the fact that glucocorticosteroid therapy stimulates hepcidin production [23]. Previous experiments have confirmed hepcidin increase by inflammatory and infectious stimuli via the interleukin 6 (IL-6) pathway [24]. Bartalena et al. have reported an increase of IL-6 in patients with SAT, which normalises following remission [25]. Elevated IL-6 was not demonstrated in any other thyroid disease manifested by thyrotoxicosis [26].

According to the manufacturer, the normal range for serum hepcidin measured by ELISA is 0.2-47.7 ng/mL. Previous research estimated a similar reference range 3.1-37.7 ng/mL for healthy population [27]. In our study, the mean ± SD values for hepcidin in healthy CS were 18.7 ± 14.3 ng/mL, being significantly lower than that in SAT patients. Based on the ROC curve for hepcidin concentration on SAT diagnosis, for the cut-off point 48.8 ng/mL, sensitivity and specificity are 52% and 95%, respectively. Thus, hepcidin might be an additional marker for SAT, also useful during treatment monitoring (decrease in hepcidin concentration reflecting the remission state) influencing therapeutic decisions (glucocorticosteroid dose tapering). Further studies on larger groups are needed to elucidate whether the magnitude of hepcidin concentration at SAT diagnosis might be useful in prediction of the risk of disease relapse. In our cohort, three patients had a relapse within three months of glucocorticosteroid therapy withdrawal. However, this number was too small for statistical evaluation.

Other markers of acute-phase inflammation assessed in the studied patients were CRP and ferritin. The CRP is a well-known and useful marker of inflammation and plays an important role in the host defence against invading pathogens [28]. Patients with SAT usually present with increased CRP levels, which normalises during remission [29]. This is rather explained with the inflammatory process, than accompanying thyroid dysfunction. However, according to Czarnywojtek et al., CRP concentration determined with a highly sensitive immunoassay is higher in hyperthyroid patients in comparison to euthyroid subjects [30]. Of note, hyperthyroid patients included in the study presented with Graves' disease or toxic nodular goitre, while only one patient with SAT was enrolled.

Both ferritin and hepcidin are acute-phase proteins as well as indicators of Fe balance [31]. We demonstrate a significant correlation between ferritin and hepcidin in SAT patients and CS. Despite improvement of all red blood cell indices following therapy of SAT, a decrease in ferritin concentration is observed. This suggests that hepcidin rise in SAT is mainly due to an acute-phase reaction, less as a reflection of accompanying Fe homeostasis imbalance. Our results are supported by the observation made on Bulgarian population that serum concentrations ferritin and hepcidin correlate significantly [27].

Lately, several studies demonstrating association between thyroid status and Fe homeostasis were published. An interesting connection between inflammatory markers and Fe balance can be derived from a study of patients with untreated different cancer types. In this group, the haemoglobin level negatively correlated with inflammatory markers (CRP, fibrinogen, IL-6, IL-1B, and tumour necrosis factor-*α*), hepcidin, ferritin, and erythropoietin. In multivariate analysis, the disease stage and IL-6 were independent predictors of haemoglobin concentration [32]. In Graves' disease patients with hyperthyroidism, Li et al. demonstrated a positive correlation between homocysteine reflecting a systemic inflammatory state and aTPO [33]. In our group of SAT patients, hepcidin_EL_ negatively correlated at the border of significance with aTPO. However, the observed relationship might be incidental and requires verification in a larger cohort of patients. Al-Hakeim et al. compared the results of thyroid function tests and markers of Fe haemostasis in children with thalassemia. They noted significantly positive correlation between TSH and hepcidin, ferritin, and Fe concentration [34]. In the study by Czarnywojtek et al., a significant positive correlation between TSH and RDW was noted in hypothyroid patients [13].

The changes in hepcidin concentration from the diagnosis of SAT until recovery occurred in parallel with changes of several other parameters. Patients with SAT had lower red blood cell indices (RBC, HGB, HCT, MCV, MCH, MCHC, RDW-CV, and Fe) but higher platelet level both if compared to CS and their own results after remission. This suggests that even the short-term acute inflammatory process accompanied by hyperthyroidism results in disturbance of Fe balance, which returns to normal following therapy. It has already been demonstrated that in the course of anaemia of chronic disease, there is a significant relationship between the platelet count and serum iron level. Kim et al. suggests that Fe deficiency in the course of anaemia of chronic diseases leads to upregulation of hepcidin due to raised inflammatory cytokines. There are two potential explanations for increased platelet count in patients with anaemia: reduced megakaryocytic Fe reserve leads to formation of megakaryocytes with raised ploidy, which can produce more platelets, and reactive thrombocytosis in inflammatory conditions caused by cytokine cascades involving IL-6 and thrombopoietin [35].

One of the observations from our study is that patients with SAT indeed present higher aTG levels, while normal aTPO and TRAb concentrations. This might be related to the destruction of thyroid cells in the course of SAT rather than chronic thyroid-oriented autoimmunity.

Studies evaluating the concentration of hepcidin in thyroid pathologies are lacking. There are only two studies already published concerning the issue of hepcidin concentration in patients with hyperthyroidism due to Graves' disease. Fischli et al. demonstrated that there is no significant difference of serum hepcidin measured by ELISA before and after treatment. However, authors noted that hepcidin levels measured by mass spectrometry were significantly lower when euthyroidism is restored if compared to the hyperthyroid state but only in males. Additionally, they observed significantly lower ferritin at follow-up than before treatment but no changes of other inflammatory markers [15]. Likewise, Lehtihet et al. did not observe significant difference in hepcidin and Fe derivatives at diagnosis of hyperthyroidism and after therapy. Only ferritin levels significantly decreased with treatment. However, the major limitation was the heterogenic study group in terms of aetiology of hyperthyroidism, follow-up period, and applied therapy, while only two patients out of 20 presented with SAT [16]. To the best of our knowledge, so far, no study concerned the issue of hepcidin in patients with SAT.

In conclusion, our results demonstrate that SAT is accompanied by significant increase in hepcidin concentration. This phenomenon reflects the state of the acute-phase inflammatory process and is accompanied by significant changes in blood count and derivatives of iron homeostasis. The remission of the disease is accompanied by a decrease in hepcidin concentration and improvement of iron homeostasis parameters. Further studies on larger groups of patients with SAT are needed to assess the potential role of hepcidin as a predictive factor of the risk of disease relapse.

## Figures and Tables

**Table 1:** Biochemical parameters in patients with subacute thyroiditis (SAT) at baseline (T0) compared to healthy control subjects (CS).

Parameter	Reference range	SAT (T0)	CS	*p* value
Hepcidin_EL_ (ng/mL)	**0.2-47.7**	**48.8 (15.9-74.5)**	**18.2 (10.2-23.3)**	**0.009** ^**1**^
WBC (×10^3^/*μ*L)	3.9-11.0	8.2 (7.1-9.1)	6.7 (5.2-8.7)	NS^1^
∗RBC (×10^6^/*μ*L)	**3.5-5.2**	4.3 ± 0.4	4.5 ± 0.3	**0.032** ^**2**^
^∗^HGB (g/dL)	**12.0-15.6**	12.3 ± 1.3	14.0 ± 1.0	**<0.001** ^**2**^
^∗^HCT (%)	**33.0-46.0**	37.1 ± 3.6	41.4 ± 2.7	**<0.001** ^**2**^
MCV (fL)	**80.0-99.0**	86.8 ± 4.2	91.1 ± 2.7	**<0.001** ^**2**^
MCH (pg)	**27.0-33.5**	**29.4 (28.2-29.7)**	**30.8 (30.0-31.8)**	**<0.001** ^**1**^
MCHC (g/dL)	**31.0-38.0**	**33.0 (32.6-33.6)**	**33.9 (33.3-34.3)**	**0.002** ^**1**^
RDW-CV (%)	**11.0-16.0**	**12.1 (11.7-12.4)**	**12.9 (12.8-13.4)**	**<0.001** ^**1**^
PLT (×10^3^/*μ*L)	**130.0-400.0**	371.4 ± 100.4	251.7 ± 53.6	**<0.001** ^**2**^
PDW (fL)	**9.0-17.0**	11.5 ± 1.9	13.1 ± 2.1	**0.013** ^**2**^
MPV (fL)	**9.0-13.0**	10.0 ± 0.9	10.8 ± 1.0	**0.009** ^**2**^
P-LCR (%)	**13.0-43.0**	25.5 ± 7.5	31.8 ± 8.2	**0.013** ^**2**^
CRP (mg/L)	**<5.0**	**38.5 (12.2-50.9)**	**1.6 (0.6-3.6)**	**<0.001** ^**1**^
ALT (U/L)	10.0-41.0	18.0 (11.0-24.0)	21.0 (14.0-24.0)	NS^1^
AST (U/L)	10.0-37.0	17.0 (13.0-20.0)	19.0 (26.0-23.0)	NS^1^
TSH (*μ*IU/mL)	**0.2-4.2**	**0.02 (0.01-0.08)**	**1.2 (1.0-1.6)**	**<0.001** ^**1**^
fT3 (pmol/L)	**3.9-6.7**	9.8 ± 4.5	4.9 ± 0.6	**<0.001** ^**2**^
fT4 (pmol/L)	**11.5-21.0**	**28.4 (23.0-39.4)**	**16.1 (14.8-18.0)**	**<0.001** ^**1**^
aTPO (IU/mL)	<34.0	14.0 (9.0-20.0)	13.0 (10.0-16.0)	NS^1^
aTG (IU/mL)	**10.0-115.0**	**33.0 (19.0-84.0)**	**13.5 (10.0-24.0)**	**0.012** ^**1**^
TRAb (IU/L)	<2.0	0.3 (0.2-0.5)	0.3 (0.2-0.4)	NS^1^
^∗^Ferritin (ng/mL)	13.0-150.0	115 (48.0-197.0)	88.0 (44.0-125.0)	NS^1^
^∗^Fe (*μ*g/dL)	**37.0-145.0**	**37.0 (29.0-70.0)**	**98.0 (70.0-139.0)**	**<0.001** ^**1**^
Creatinine (mg/dL)	0.5-1.2	0.6 (0.5-0.7)	0.7 (0.70.8)	NS^1^

Values are expressed as median (IQR) for nonparametric tests and mean ± SD for parametric tests. ^1^Mann–Whitney *U* test; ^2^independent *T*-test. ^∗^Parameters with different reference ranges in men and women, test performed only in the female subgroup. NS: nonsignificant; WBC: white blood cells; RBC: red blood cells; HGB: haemoglobin; HCT: haematocrit; MCV: mean corpuscular volume; MCH: mean corpuscular haemoglobin; MCHC: mean corpuscular haemoglobin concentration; RDW-CV: red blood cell distribution width-coefficient of variation; PLT: platelets; PDW: platelet distribution width; MPV: mean platelet volume; P-LCR: platelet larger cell ratio; CRP: C-reactive protein; ALT: alanine aminotransferase; AST: aspartate aminotransferase; TSH: thyroid-stimulating hormone; fT3: free triiodothyronine; fT4: free thyroxine; aTPO: anti-thyroid peroxidase antibody; aTG: anti-thyroglobulin antibody; TRAb: thyrotropin receptor antibody; Fe: iron.

**Table 2:** Biochemical parameters in patients with subacute thyroiditis (SAT) at baseline (T0) compared to the values obtained following complete remission of the disease (T1). Only parameters demonstrating statistically significant differences are presented.

Parameter	Reference range	SAT baseline (T0)	SAT follow-up (T1)	*p* value
Hepcidin_EL_ (ng/mL)	0.2-47.7	48.8 (15.9-74.5)	4.0 (1.2-10.0)	<0.001^1^
WBC (×10^3^/*μ*L)	3.9-11.0	8.1 ± 1.6	6.5 ± 1.6	<0.001^2^
^∗^RBC (×10^6^/*μ*L)	3.5-5.2	4.3 ± 0.4	4.6 ± 0.3	0.002^2^
^∗^HGB (g/d)	12.0-15.6	12.2 ± 1.3	13.5 ± 0.9	<0.001^2^
^∗^HCT (%)	33.0-46.0	36.9 ± 3.6	39.8 ± 2.2	0.003^2^
MCH (pg)	27.0-33.5	29.4 (28.2-29.7)	29.3 (28.5-30.7)	0.004^1^
MCHC (g/dL)	31.0-38.0	33.0 (32.6-33.6)	33.9 (33.3-34.5)	0.007^1^
RDW-CV (%)	11.0-16.0	12.1 (11.7-12.4)	13.5 (12.7-14.5)	<0.001^1^
PLT (×10^3^/*μ*L)	130.0-400.0	371.2 ± 103.0	276.0 ± 73.0	<0.001^2^
PDW (fL)	9.0-17.0	11.5 ± 1.9	12.4 ± 2.3	0.001^2^
MPV (fL)	9.0-13.0	10.0 ± 0.9	10.4 ± 1.0	<0.001^2^
P-LCR (%)	13.0-43.0	25.4 ± 7.7	28.2 ± 8.2	<0.001^2^
CRP (mg/L)	<5.0	38.5 (12.2-50.9)	1.0 (0.5-2.0)	<0.001^1^
TSH (*μ*U/mL)	0.2-4.2	0.02 (0.01-0.1)	1.2 (0.7-2.0)	<0.001^1^
fT3 (pmol/L)	3.9-6.7	9.9 ± 4.6	4.9 ± 0.6	<0.001^2^
fT4 (pmol/L)	11.5-21.0	28.4 (23.0-39.4)	15.2 (14.6-16.8)	<0.001^1^
TRAb (IU/L)	<0.2	0.3 (0.2-0.5)	0.2 (0.1-0.3)	0.015^1^
^∗^Ferritin (ng/mL)	13.0-150.0	115 (48.0-197.0)	34.5 (25.0-43.0)	<0.001^1^
^∗^Fe (*μ*g/dL)	37.0-145.0	37.0 (29.0-70.0)	98.5 (65.0-111.0)	0.001^1^

Values are expressed as median (IQR) for nonparametric tests and mean ± SD for the parametric test. ^1^Wilcoxon test; ^2^dependent *T*-test, ^∗^ parameters with different reference ranges in men and women, test performed only in the female subgroup. WBC: white blood cells; RBC: red blood cells; HGB: haemoglobin; HCT: haematocrit; MCH: mean corpuscular haemoglobin; MCHC: mean corpuscular haemoglobin concentration; RDW-CV: red blood cell distribution width-coefficient of variation; PLT: platelets; PDW: platelet distribution width; MPV: mean platelet volume; P-LCR: platelet larger cell ratio; CRP: C-reactive protein; TSH: thyroid-stimulating hormone; fT3: free triiodothyronine; fT4: free thyroxine; TRAb: thyrotropin receptor antibody; Fe: iron.

## Data Availability

The data used to support the findings of this study are included within the article and within the supplementary information files. Requests for access to any additional data should be made to the corresponding author.

## Supplementary Materials

Supplementary Materials Supplementary Table 1: biochemical parameters demonstrating statistically significant difference between the patients with subacute thyroiditis (SAT) after remission and the control group (CS). Supplementary Table 2: univariate analyses for correlation between hepcidin_EL_ and selected parameters in patients with subacute thyroiditis (SAT) at baseline (T0) and control subjects (CS). Supplementary Figure 1: the receiver operating characteristic (ROC) curve for the level of hepcidin_EL_ of the patients with subacute thyroiditis (SAT) at the moment of diagnosis (AUC = 0.735, *p* = 0.009). Supplementary Figure 2: the sensitivity and specificity of the hepcidin_EL_ level in patients with subacute thyroiditis (SAT) at the moment of diagnosis. The crossing point of proportion (hepcidinn_EL_ = 22.5 ng/mL) is determined at sensitivity (0.66) and specificity (0.71). Supplementary Figure 3: Spearman's correlation at the moment of diagnosis (SAT T0) for (a) hepcidin_EL_ and C-reactive protein (CRP) (*r* = 0.614, *p* = 0.003), (b) hepcidin_EL_ and ferritin (*r* = 0.815, *p* < 0.001), and (c) hepcidin_EL_ and anti-thyroid peroxidase antibodies (aTPO) (*r* = -0.491, *p* = 0.024). Supplementary Figure 4: Spearman's correlations in the control group (CS) for (a) hepcidin_EL_ and ferritin (*r* = 0.837, *p* < 0.001) and (b) hepcidin_EL_ and iron (Fe) (*r* = 0.540, *p* = 0.017).
