# Supplementary material for: Hepcidin and Iron Homeostasis in Patients with Subacute Thyroiditis and Healthy Subjects
Source: Mediators Inflamm. 2019 Feb 27;2019:5764061. doi: 10.1155/2019/5764061 (PMC6415316; doi:10.1155/2019/5764061)

**Supplementary Table 1. Biochemical parameters demonstrating statistically significant difference between the patients with subacute thyroiditis (SAT) after remission and control group (CS).**

| **Parameter** | **Reference rage** | **SAT (T1)** | **CS** | **p-value** |
| --- | --- | --- | --- | --- |
| **Hepcidin_EL,_ [ng/mL]** | **0.2 - 47.7** | **4.0 [1.2-10.0]** | **18.2 [10.2-23.3]** | **0.007^1^** |
| **MCV, [fl]** | **80.0 - 99.0** | **87.2 ± 3.7** | **91.1 ± 2.7** | **<0.001^2^** |
| **MCH, [pg]** | **27.0 - 33.5** | **29.6 ± 1.4** | **30.9 ± 1.4** | **0.004^2^** |
| **RDW-CV, [%]** | **11.0 - 16.0** | **13.7 ± 1.3** | **13.0 ± 0.7** | **0.040^2^** |
| ***Ferritin, [ng/mL]** | **13.0 - 150.0** | **34.4 [25.0-43.0]** | **88.0 [44.0-125.0]** | **0.003^1^** |

Values are expressed as median [IQR] for nonparametric tests and mean ± SD for parametric test

^1^Mann-Whitney U-test, ^2^Independet T-test,

* parameters with different reference range in men and women; test performed only in the female subgroup,

MCV - mean corpuscular volume, MCH - mean corpuscular haemoglobin, RDW-CV - red blood cell distribution width, SD - standard deviation.

**Supplementary Table 2. Univariate analyses for correlation between hepcidin_EL_ and selected parameters in patients with subacute thyroiditis (SAT) at baseline (T0) and control subjects (CS).**

| **Parameter** | **SAT T0** | **p-value** | **CS** | **p-value** |
| --- | --- | --- | --- | --- |
| WBC, x 10^3/µl | -0.009^1^ | NS | 0.063^1^ | NS |
| *HGB, [g/d] | -0.297^1^ | NS | 0.310^2^ | NS |
| CRP, [mg/L] | **0.614^1^** | **0.003** | 0.087^1^ | NS |
| *Ferritin, [ng/mL] | **0.815^1^** | **<0.001** | **0.837^1^** | **<0.001** |
| *Fe, [µg/dL] | -0.419^1^ | NS | **0.459^2^** | **0.048** |

***** parameters with different reference range in men and women; test performed only in the female subgroup, ^1^Spearman’s correlation coefficient, ^2^Pearson’s correlation coefficient, NS - non-significant, WBC - white blood cells, HGB - haemoglobin, CRP - C-reactive protein, Fe - iron.

**Supplementary Figure 1. The receiver operating characteristic (ROC) curve for the level of hepcidin_EL_ of the patients with subacute thyroiditis (SAT) at the moment of diagnosis (AUC=0.735, p=0.009).**


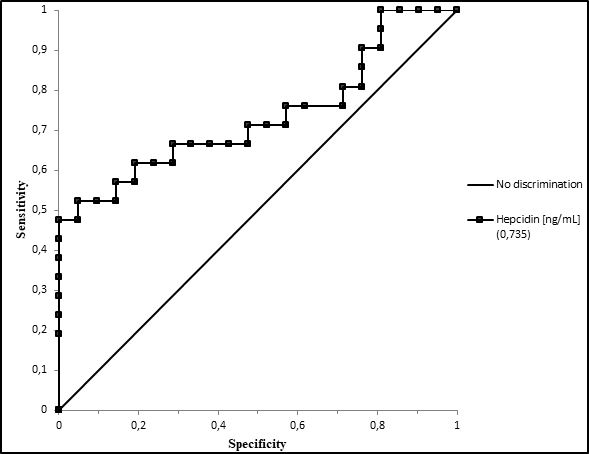


**Supplementary Figure 2. The sensitivity and specificity of hepcidin_EL_ level in patients with subacute thyroiditis (SAT) at the moment of diagnosis. The crossing point of proportion (hepcidin_EL_ = 22.5 ng/mL) is determined at sensitivity (0.66) and specificity (0.71).**


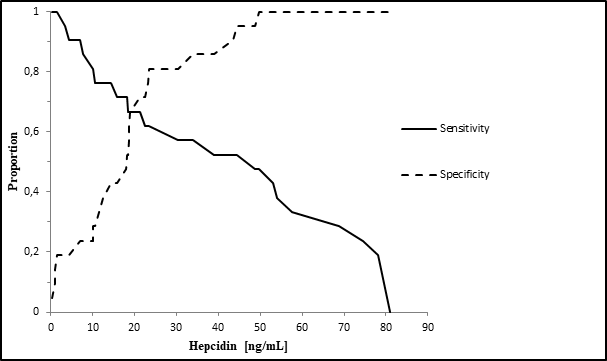


**Supplementary Figure 3. The Spearman’s correlation at the moment of diagnosis (SAT T0) for: a) hepcidin_EL_ and C-reactive protein (CRP), (r=0.614, p=0.003), b) hepcidin_EL_ and ferritin, (r=0.815, p<0.001) c) hepcidin_EL_ and anti-thyroid peroxidase antibodies (aTPO), (r= -0.491, p=0.024).**


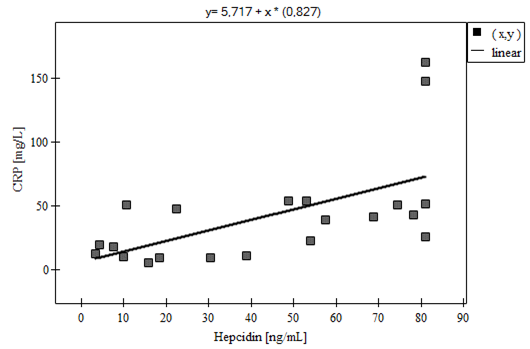


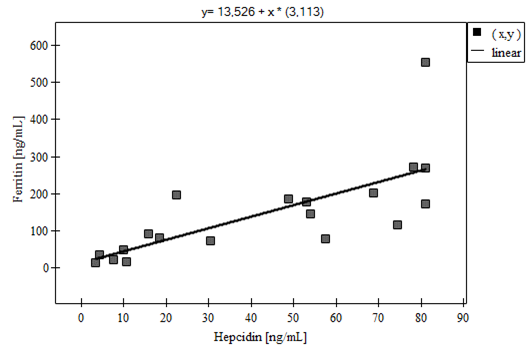

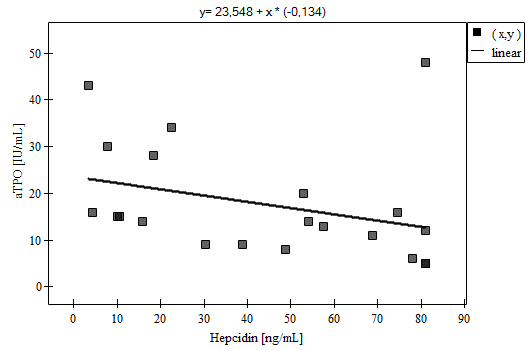


**Supplementary Figure 4. The Spearman’s correlations in the control group (CS) for: a) hepcidin_EL_ and ferritin, (r=0.837, p<0.001) b) hepcidin_EL_ and iron (Fe), (r=0.540, p=0.017).**


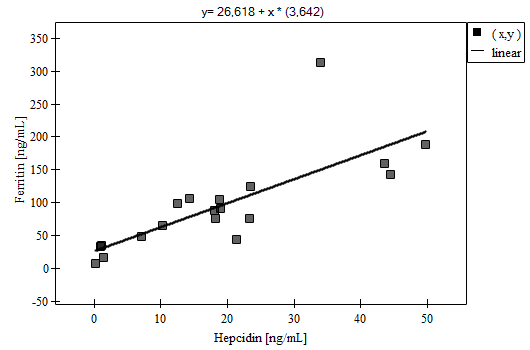

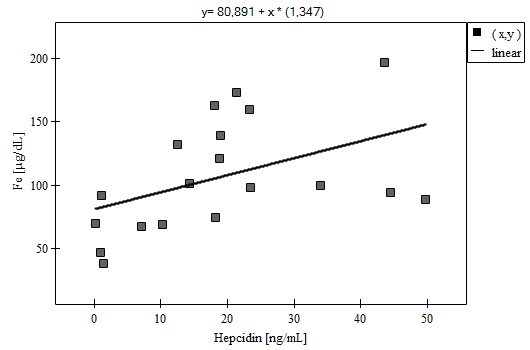

Supplement: Supplementary Materials — Supplementary Table 1: biochemical parameters demonstrating statistically significant difference between the patients with subacute thyroiditis (SAT) after remission and the control group (CS). Supplementary Table 2: univariate analyses for correlation between hepcidinEL and selected parameters in patients with subacute thyroiditis (SAT) at baseline (T0) and control subjects (CS). Supplementary Figure 1: the receiver operating characteristic (ROC) curve for the level of hepcidinEL of the patients with subacute thyroiditis (SAT) at the moment of diagnosis (AUC = 0.735, p = 0.009). Supplementary Figure 2: the sensitivity and specificity of the hepcidinEL level in patients with subacute thyroiditis (SAT) at the moment of diagnosis. The crossing point of proportion (hepcidinnEL = 22.5 ng/mL) is determined at sensitivity (0.66) and specificity (0.71). Supplementary Figure 3: Spearman's correlation at the moment of diagnosis (SAT T0) for (a) hepcidinEL and C-reactive protein (CRP) (r = 0.614, p = 0.003), (b) hepcidinEL and ferritin (r = 0.815, p < 0.001), and (c) hepcidinEL and anti-thyroid peroxidase antibodies (aTPO) (r = -0.491, p = 0.024). Supplementary Figure 4: Spearman's correlations in the control group (CS) for (a) hepcidinEL and ferritin (r = 0.837, p < 0.001) and (b) hepcidinEL and iron (Fe) (r = 0.540, p = 0.017). [file 5764061.f1.docx]
